# The synergistic efficacy of hydroxychloroquine with methotrexate is accompanied by increased erythrocyte mean corpuscular volume

**DOI:** 10.1093/rheumatology/keab403

**Published:** 2021-05-04

**Authors:** Muhammad Ruhul Amin Shipa, Su-Ann Yeoh, Andrew Embleton-Thirsk, Dev Mukerjee, Michael R Ehrenstein

**Affiliations:** Centre for Rheumatology, Division of Medicine, University College London, London, UK and Rayne Institute, 5 University St, Bloomsbury, London WC1E 6JF; Centre for Rheumatology, Division of Medicine, University College London, London, UK and Rayne Institute, 5 University St, Bloomsbury, London WC1E 6JF; Comprehensive Clinical Trials Unit, University College London, Institute of Clinical Trials & Methodology, Faculty of Population Health Sciences, 90 High Holborn, Holborn, London WC1V 6LJ; Department of Rheumatology, North Middlesex University Hospital NHS Trust, London, UK and Sterling Way, London N18 1QX; Centre for Rheumatology, Division of Medicine, University College London, London, UK and Rayne Institute, 5 University St, Bloomsbury, London WC1E 6JF

**Keywords:** MTX, HCQ, mean corpuscular change, biomarker, synergistic

## Abstract

**Objectives:**

To determine whether concomitant HCQ modulates the increase in erythrocyte mean corpuscular volume (MCV) caused by MTX therapy, and whether this is associated with improved clinical response in RA.

**Methods:**

A retrospective observational analysis was conducted on two independent hospital datasets of biologic-naïve, early-RA patients who started oral MTX. Baseline characteristics, DAS28-ESR and monthly MCV after starting MTX were obtained. Conventional and machine-learning statistical approaches were applied to the discovery cohort (Cohort 1, 655 patients) and results validated using Cohort 2 (225 patients).

**Results:**

HCQ therapy with MTX was associated with a 2-fold increase in the likelihood of response defined in this study as clinical remission or low disease activity at 6 months (*P* <0.001). The improved clinical outcome of combination HCQ and MTX therapy was associated with an accelerated rise in MCV from 2 months after commencing therapy. The increase in MCV at 3 months was equivalent to the contemporaneous reduction in the DAS (DAS28-ESR) in predicting clinical response at 6 months. Using latent class mixed modelling, five trajectories of MCV change over 6 months from baseline were identified. The odds ratio of response to treatment was 16.2 (95% CI 5.7, 46.4, *P* <0.001) in those receiving combination therapy classified within the MCV elevation >5 fl class, which contained the most patients, compared with MTX alone.

**Conclusion:**

Our data provide mechanistic insight into the synergistic clinical benefit of concomitant HCQ with MTX, boosting the rise in MCV, which could serve as a companion biomarker of treatment response.

**Rheumatology key messages**

- Concomitant HCQ with MTX is associated with an improved clinical response in RA.
- HCQ appears to act synergistically with MTX to boost the increase in erythrocyte mean corpuscular volume.
- Mean corpuscular volume can act as an early indicator of response to MTX with or without HCQ.

## Introduction

MTX remains the most frequently used first-line DMARD for RA and is the anchor drug for the treat-to-target strategy aiming to achieve clinical remission [1]. MTX monotherapy has recently emerged as the first-line treatment option for RA as international guidelines [1, 2] veer away from initial combination DMARD therapy supported by several recent studies (including an indirect comparison meta-analysis) [3, 4]. However, a number of publications have supported the use of concomitant HCQ with MTX [5, 6]. In accordance with legacy National Institute for Health and Care Excellence recommendations [7], many patients in the UK were started on initial combination therapy, which in our practice was predominantly MTX and HCQ until the 2018 update [8].

There are conflicting reports relating to how HCQ affects the pharmacodynamics of MTX. HCQ was found to increase the exposure to MTX [9], though others suggested it reduced the absorption of MTX resulting in lower bioavailability [10]. MTX undergoes rapid intracellular uptake after absorption [11, 12]. One effect of MTX is to increase erythrocyte mean corpuscular volume (MCV), a component of the full blood count, which is routinely measured during treatment. An association between MCV and clinical response to MTX has been described [13]. Thus, the impact of MTX on MCV may be useful and readily available surrogate to address whether HCQ affects the bioavailability of MTX. To provide insight into the potential synergy between these two DMARDs, we documented the longitudinal change in erythrocyte MCV after commencing MTX with or without HCQ and its relationship with clinical outcome.

## Methods

A real-world data analysis was performed on adult, biologic-naïve, early RA patients (according to 2010 ACR/EULAR classification criteria) [14] who had started oral MTX from January 2010 to December 2019 at University College London Hospital (discovery cohort, Cohort 1) and at North Middlesex University Hospital (validation cohort, Cohort 2). Patients with known folate or B12 deficiency, active thyroid dysfunction or myelodysplastic syndrome were excluded due to the potential impact upon MCV.

Data were extracted from the hospitals’ clinical database in June 2020. Demographics and baseline disease characteristics (age, gender, race, disease duration, haemoglobin, CRP, smoking status, antibody status) were recorded. Patients were defined as seropositive if they were positive for RF or anti-CCP antibodies according to the local laboratory normal range. DAS28-ESR disease outcome scores were obtained at baseline, and at 3 and 6 months. MCV was collected at baseline and monthly for 6 months. Concomitant use of oral prednisolone and conventional synthetic DMARDs, such as HCQ, SSZ and LEF, were recorded.

Response was defined as attainment of remission (DAS28-ESR ≤2.6) or low disease activity (DAS28-ESR 2.6–3.2) at 6 months after commencing MTX and no initiation of additional DMARDs within 6 months of MTX initiation.

### Statistics

Statistical analysis was performed using R software version 4.0.2 for Mac OS (R Foundation for Statistical Computing, Vienna, Austria).

For time-dependent variables such as MCV change from baseline, monthly area under the receiver operating characteristics (AUROC) to predict MTX response at 6 months were calculated using Cohort 1. Following univariate logistic regression, to select predictors for the final multiple logistic regression model, the least absolute shrinkage and selection operator penalized regression method was applied. The accuracy of this model was tested using Cohort 2. The effects of the variables were further tested on Cohort 1 using different methods of supervised machine learning for classification. A linear mixed model was fitted using random-effect for individual patients and fixed-effect for follow-up time intercepting with treatment outcomes and types of treatments, to examine the longitudinal change of MCV (pairwise *post* *hoc* comparisons by Bonferroni correction). Finally, latent class mixed models were used to investigate trajectories of MCV change from baseline to 6 months with mixed-effect models. The characteristics of the patients between latent classes were tested by non-parametric testing. (Further statistical details are provided in Supplementary Methods, available at *Rheumatology* online.)

### Ethics approval

Ethical approval was not required by the National Health Service Research Ethics Committee.

## Results

### Baseline characteristics of the two cohorts

Selection of patients with their baseline demographics and disease characteristics are summarized in supplementary Fig. S1 and Table S1, available at *Rheumatology* online. In Cohort 1 55% (358/655) and in Cohort 2 58% (130/225) achieved either DAS28-ESR remission or low disease activity at 6 months. Some 34% (224/655) and 35% (78/225) of patients achieved remission at 6 months in Cohort 1 and Cohort 2, respectively.

### Identification of predictors of response to MTX at 6 months

**Fig. 1:**
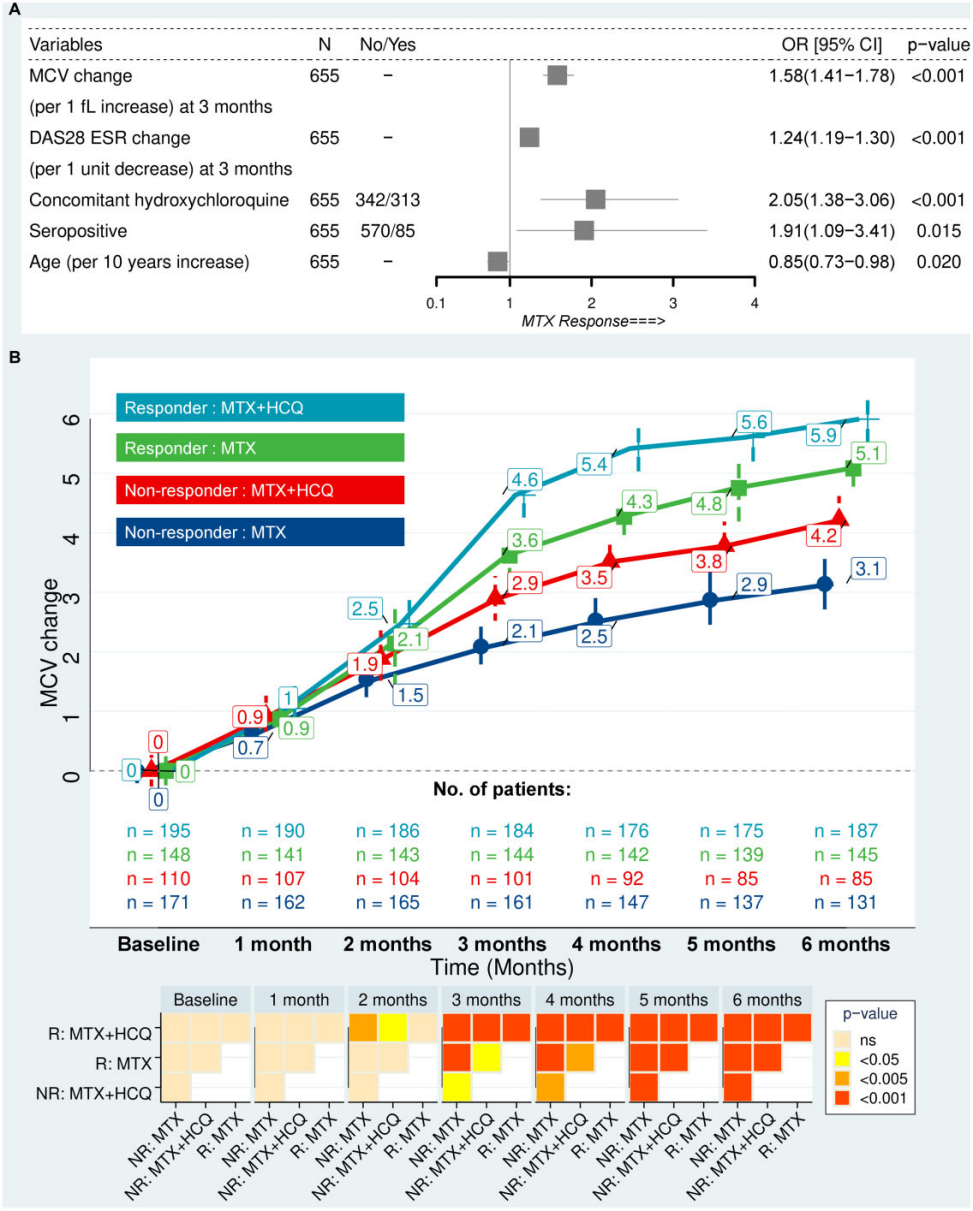
HCQ boosts the MCV elevation caused by MTX, which is associated with improved clinical outcome (Cohort 1) (**A**) Multiple logistic regression using variables selected by least absolute shrinkage and selection operator regression to predict MTX response (defined as remission or low disease activity, DAS28-ESR < 3.2) at 6 months. (**B**) Changes in MCV after initiation of MTX with or without HCQ stratified by responder (R) *vs* non-responder (NR) status. Linear mixed model was used to estimate mean changes of MCV at each time point from baseline. Mean changes (shown in the boxes) with 95% CI are shown in the plot. A heatmap demonstrating *P*-values of pairwise comparisons of MCV increase (at monthly intervals) between the treatment and response groups is also shown. MCV: mean corpuscular volume; OR: odds ratio.

We first determined the relationship between the monthly changes in MCV after commencing MTX, and clinical outcome at 6 months, in particular attainment of remission or low disease activity. AUROC of monthly change in MCV to predict MTX response at 6 months are shown in supplementary Fig. S2, available at *Rheumatology* online. MCV change at 3 months was the earliest time point where the AUROC was >0.70 and therefore was selected for the logistic regression model to evaluate MCV as a predictor of remission at 6 months and its interaction with other variables including concomitant HCQ. Univariate logistic regression of the variables was performed (supplementary Fig. S3, available at *Rheumatology* online). Five variables were retained (Fig. 1A) for the final model (AUROC 0.82, McFadden’s R Square 0.44). A rise in MCV (by 1 fl from baseline) and reduction of DAS28-ESR score (by 1 unit from baseline) at 3 months increased the likelihood of clinical response at 6 months by 58% (95% CI 41%, 78%, *P* < 0.001) and 24% (95% CI 19%, 30%, *P* < 0.001), respectively. Concomitant HCQ and positive antibody status predicted a favourable response to MTX at 6 months, with an odds ratio (OR) of 2.05 (95% CI 1.38, 3.06, *P*<0.001) and 1.91 (95% CI 1.09, 3.41, *P=*0.015), respectively. In contrast, the likelihood of MTX response was reduced with increasing age, OR of 0.85 (95% CI 0.73, 0.98, *P*=0.02). The final selected model revealed an accuracy of 81% (95% CI 76%, 86%) with respect to predicting clinical response at 6 months, when the model generated from Cohort 1 (training set) was tested on Cohort 2 (specificity 90% and sensitivity 70%). The model accuracy reduced to 70% when MCV change at 3 months was removed from the model but was unaffected when DAS28-ESR was removed (supplementary Table S2, available at *Rheumatology* online).

To account for potentially different treatment strategies for patients according to seropositivity, we analysed the treatment response stratified by antibody status. Propensity scores were estimated for each patient using logistic regression adjusted for age, disease duration, sex, smoking status, baseline DAS, CRP at baseline, dose of MTX, and other DMARDs and their dose. Seropositivity still showed a favourable effect on response, with an OR of 1.51 (95% CI 1.12, 2.36, *P* =0.027), and is consistent with previous findings [15].

After testing different machine-learning approaches (supplementary Fig. S4A and B, available at *Rheumatology* online) the top two performing methods—support vector machine and logistic regression—were selected. MCV change at 3 months had the highest importance score using the support vector machine method (supplementary Fig. S5A, available at *Rheumatology* online), whereas DAS28-ESR was the most important followed by MCV change at 3 months using logistic regression (supplementary Fig. S5B, available at *Rheumatology* online).

The optimal cut-point for MCV increment was 3.5 fl, which predicted clinical response at 6 months (95% CI 3.5, 3.6) with a sensitivity of 62% and specificity of 81% (supplementary Fig. S6, available at *Rheumatology* online). A raised MCV of ≥3.6 fl was not associated with increased adverse drug reactions, irrespective of whether this was combined with HCQ (supplementary Figs S7 and S8, available at *Rheumatology* online).

### HCQ boosts the increase in MCV by MTX and is associated with improved clinical outcome

Changes in MCV after initiation of MTX with or without HCQ, stratified by their clinical response status, are detailed in Fig. 1B (Cohort 1). Responding patients on combination therapy (MTX and HCQ) had an accelerated rise in MCV, compared with non-responders, from month 2 (Fig. 1B). Patients responding to MTX monotherapy had a significant difference in MCV change compared with MTX monotherapy non-responders from month 3. A rise in MCV also occurred in patients who did not respond to the combination compared with those who did not respond to MTX monotherapy, though this change in MCV was less than with responding patients. Similar trends were observed in Cohort 2 (supplementary Fig. S9A and B, available at *Rheumatology* online).

**Fig. 2:**
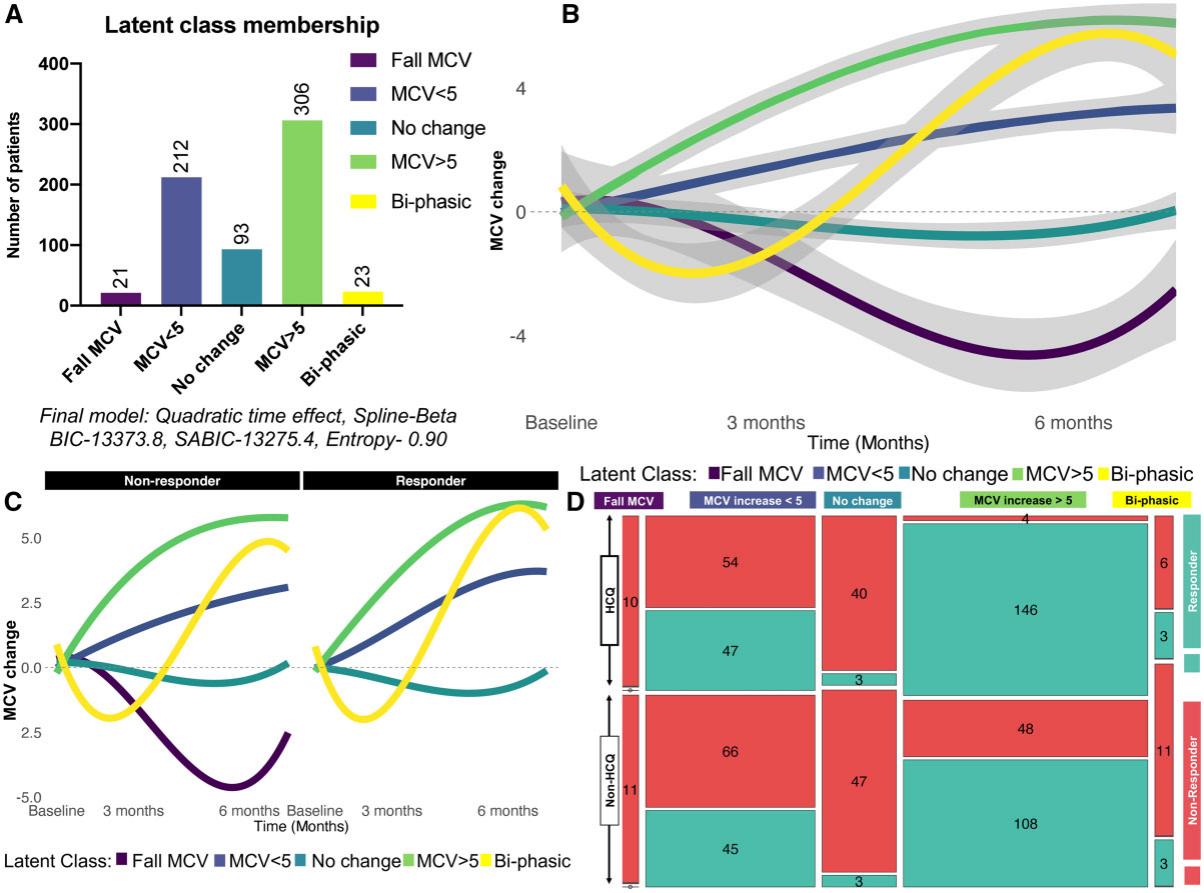
Trajectories of latent class of MCV change over 6 months after initiation of MTX (Cohort 1) The five classes are: (i) Fall MCV: reduction in MCV; (ii) MCV <5: MCV increase <5 fl; (iii) No change: no MCV change; (iv) MCV >5: MCV increase >5 fl; and (v) Bi-phasic: initial reduction in MCV followed by an increase, from baseline. The number of patients in each class (**A**) and their MCV trajectories (**B**) are shown. (**C**) Stratification of the trajectories of the five-class model between non-responders and responders. (**D**) Mosaic plot illustrating cross-sectional distribution of patients from each latent class stratified by treatment response and treatment. MCV: mean corpuscular volume.

Five latent classes were identified in Cohort 1 using latent class mixed modelling (Fig. 2) and named based on changes in MCV measured in fl. The most prevalent class was the ‘MCV increase >5’ group (*N* = 306, 47%), followed by the ‘MCV increase <5’ group (*N* = 212, 32%) (Fig. 2A and B). A much smaller class, ‘Fall in MCV’ (*N* = 21, 3%), was not present in the responder group (Fig. 2C). Four latent classes were identified when the analysis was confined to patients receiving MTX monotherapy: the ‘Fall in MCV’ class was not present. The same five latent classes remained when patients receiving the MTX and HCQ as a combination were analysed separately. In the ‘MCV increase >5’ class, there were five times more responders compared with non-responders (254 responders *vs* 52 non-responders, Fisher’s exact test *P<*0.001) (supplementary Fig. S10, available at *Rheumatology* online). In contrast, the vast majority of patients in the ‘No change’ class were non-responders (87 non-responders *vs* 6 responders, Fisher’s exact test *P<*0.001) and no responders were contained within the ‘Fall in MCV’ class. The ‘MCV increase >5’ class contained a significantly larger proportion of patients who responded to the combination of HCQ and MTX (97%; 146 responders *vs* 4 non-responders) compared with the proportion responding to MTX monotherapy (69%; 108 responders *vs* 48 non-responders), with a p-value of <0.001 (Fig. 2D).

The OR of response to treatment was 16.2 (95% CI 5.7, 46.4, *P<*0.001) in those receiving combination therapy classified within the ‘MCV increase >5’ class compared with MTX monotherapy.

The same five latent class model was applied to Cohort 2. The ‘Fall MCV’ class was not observed (supplementary Fig. S11A and B, available at *Rheumatology* online]. The ‘MCV increase 5–7.5’ and ‘MCV increase <2.5’ contained the majority of the patients, 95 (42%) and 85 (38%), respectively (supplementary Fig. S11B, available at *Rheumatology* online). The few patients in the ‘MCV increase >7.5’ class all responded to therapy (supplementary Fig. S11C, available at *Rheumatology* online). In the ‘MCV increase 5–7.5’ class, there was a higher number of responders (*N* = 81, *vs* 14 non-responders), and in the ‘MCV increase <2.5’ class, a higher number of non-responders (*N* = 66, *vs* 19 responders) were present (supplementary Fig. S11D, available at *Rheumatology* online), with a *P-*value of <0.001.

## Discussion

These data reveal HCQ as a key determinant of the relationship between increase in MCV and clinical response to MTX. The greatest increase in MCV occurred with concomitant HCQ, which was associated with better clinical outcome compared with MTX monotherapy. Using latent class modelling, a vastly greater proportion of responders to combination HCQ and MTX, compared with the proportion responding to MTX monotherapy, characterized the class with an MCV increase >5 fl. The accelerated increase in MCV, which occurred as early as month 2 after starting treatment with the combination, provides insight into the mechanism underpinning the therapeutic synergism between these two DMARDs as reported by others [5, 6].

The DAS at 3 months is well established as a critical therapeutic decision point and a strong predictor of MTX response at 6 months [16]. The early increase in MCV following MTX therapy represents an objective and routinely available measure that could supplement clinical assessment and facilitate rapid optimization of therapy. Its accuracy as a biomarker to predict remission or low disease activity at 6 months was greatest when concomitant HCQ was prescribed with MTX. Our data identify the threshold increase in MCV that would indicate increased likelihood of attainment of low disease activity or remission, providing a semi-quantitative tool to guide therapeutic decision making. It would be interesting to explore whether change in erythrocyte MCV can predict sustained clinical response beyond 6 months. Inadequate changes in MCV could also prompt clinicians to address treatment adherence. Its value as a biomarker is emphasized during the current pandemic, where objective clinical assessments have been more difficult to perform due to a restriction on face-to-face visits [17]. Indeed, these restrictions during the pandemic may influence hospitals to increase the number of virtual visits for patients with long-term conditions even after the pandemic has resolved.

The strength of our study is the addition of a validation cohort (Cohort 2) from a separate hospital to test the results found in our discovery cohort (Cohort 1). The folic acid dosage regimen of 5 mg once daily, five times a week, is identical in both cohorts, reducing the effect of this as a potential confounder. There are limitations due to the retrospective nature of our study. Alcohol intake, diet and some concurrent medication can be an important confounder of MCV change. All patients are advised to restrict their alcohol intake when starting MTX, but alcohol consumption has not been taken into account in our analysis due to the paucity of data on baseline alcohol consumption or changes in alcohol consumption after starting MTX. Similarly, concomitant use of medications that can influence MCV (e.g. proton pump inhibitors [18]) or dietary habits were not accounted for in the analysis. Use of i.m. CS was not available in our dataset, which may impact the short-term response. None of these factors is likely to fully explain the apparent benefit of concomitant HCQ with MTX, and the association with clinical response and a rise in MCV. Treatment adherence is an important confounder that should be addressed in future studies. These results should be reproduced in cohorts with lower rates of RF and/or CCP positivity than the frequency of 86% we have reported.

Our study supports the therapeutic benefit of concomitant HCQ with MTX and also identifies MCV change as an accurate predictor of treatment response to MTX. This MCV change by MTX is accentuated by the addition of HCQ, providing a potential explanation for their therapeutic synergism. Further prospective studies are required to confirm these findings as well as research designed to explore the basis for the apparent interaction between MCV change, MTX/HCQ combination therapy and clinical response.

## Supplementary Material

keab403_supplementary_data
