# Supplementary material for: The synergistic efficacy of hydroxychloroquine with methotrexate is accompanied by increased erythrocyte mean corpuscular volume
Source: Rheumatology (Oxford). 2021 May 4;61(2):787–93. doi: 10.1093/rheumatology/keab403 (PMC8824424; doi:10.1093/rheumatology/keab403)
Supplement: keab403_supplementary_data [file keab403_supplementary_data.docx]

**SUPPLEMENTARY METHODS:**

Latent class mixed models were used to investigate trajectories of MCV change from baseline to six months with mixed-effect models, and within-individual correlation along with fixed and random individual-level interception and slopes. The final model of latent class mixed models was selected by lowest Bayesian information criteria (BIC) with quadratic time effect and beta spline using lcmm function from the lcmm(1) package for R after testing various time effects (linear, quadratic and cubic), splines (linear, beta, splines and 5-quant-splines) and the number of classes (K=1-10). The MCV change was adjusted for age at baseline, disease duration, gender, ethnicity, methotrexate dose at three months, prednisolone dose at three months, baseline hemoglobin, baseline estimated glomerular filtration rate, and concomitant disease modifying agent (methotrexate, hydroxychloroquine or sulfasalazine). *Model accuracy was assessed by the average of the maximum posterior probability of assignments (APPA) – 1. Fall MCV: 0.966, 2. MCV <5: 0.914, 3. No change: 0.882, 4. MCV>5: 0.934, and 5. Bi-phasic: 0.917.* For latent class mixed modelling, missing values were imputed using multivariate imputation by chained equations of the Markov chain Monte Carlo method under the ‘missing at random’ assumption (2).

**SUPPLEMENTARY TABLES AND FIGURES:**

**Supplementary Figure 1: CONSORT diagram.** Number of patients who were started on oral methotrexate from the two cohorts. In both cohorts, the folic acid dose was 5mg once daily, 5 times a week.

**Supplementary Table 1: Baseline characteristics of biologic-naïve patients (N=880) with rheumatoid arthritis before commencing oral methotrexate.**

|  | Total  N = 880 | Cohort 1  (Discovery cohort)  N = 655^a^ | Cohort 2  (Validation cohort)  N = 225^a^ |
| --- | --- | --- | --- |
| Age, years | 49·3 (13·1) | 48·7 (12·3) | 49·2 (13·2) |
| Female, n (%) | 613 (69.7) | 448 (68.4) | 165 (73.3) |
| Caucasian, n (%) | 517 (58.8) | 390 (59.5) | 127 (56.4) |
| Seropositive *^b^*, n (%) | 756 (85.9) | 570 (87) | 186 (82.6) |
| Rheumatoid factor positive, n (%) | 636 (72.3) | 483 (73.7) | 153 (68%) |
| Anti-CCP positive, n (%) | 621(70.6) | 471(71.9) | 150(66.7) |
| Disease duration, months | 7 (4) | 7 (4) | 8 (2) |
| Tender joint count  *Missing, n (%) ^c^* | 9 (3)  *64 (7.3)* | 8 (3)  *42 (6.4)* | 10 (4)  *22 (9.8)* |
| Swollen joint count  *Missing, n (%)* | 5 (2)  *42 (4·7)* | 5 (2)  *31 (4.7)* | 6 (3)  *11 (4·9)* |
| DAS28-ESR  *Missing, n (%)* | 4·63 (0·72)  *28 (3·2)* | 4·68 (0·91)  *19 (3)* | 4·49 (0·75)  *9 (4)* |
| C-reactive protein, mg/L  *Missing, n (%)* | 15·1 (13·3)  *73 (8.3)* | 16·1 (14·4)  *56 (8.5)* | 17·3 (13·2)  *17 (7.5)* |
| eGFR, ml/min/1.73 m^2^  *Missing, n (%)* | 80 (13)  *72 (8.2)* | 81 (12)  *51 (7·8)* | 74 (13)  *21 (9.3)* |
| Haemoglobin, g/L | 119 (17) | 120 (13) | 117 (17) |
| MCV, fL | 90·9 (2·9) | 90·9 (3·0) | 89·8 (2·7) |
| Concomitant prednisolone, n (%) | 374 (42.5) | 275 (42) | 99 (44) |
| Prednisolone dose, (mg/day) | 4.9(6.1) | 4.7(6.0) | 5.2(6.2) |
| Treatment, n (%) |  |  |  |
| Methotrexate monotherapy, n (%) | 393 (44.7) | 319 (48.7) | 74 (32.9) |
| Methotrexate and  hydroxychloroquine, n (%) | 441 (50.1) | 305 (46.6) | 136 (60.4) |
| Methotrexate and sulfasalazine, n (%) | 30 (3.4) | 23 (3.5) | 7 (3.1) |
| Methotrexate, sulfasalazine and |  |  |  |
| Hydroxychloroquine, n (%) | 16 (1.8) | 8 (1.2) | 8 (3.6) |
| Active smoker, n (%)  *Missing, n (%)* | 196 (31.7) *^d^*  *262 (29.7)* | 142 (30.5) *^d^*  *190 (29)* | 54 (35.8) *^d^*  *74 (32.9)* |
| ^a^*Statistics presented: mean (SD); n = number (% within each cohort, unless otherwise specified)*  ^b^ Seropositive to either rheumatoid factor or anti-CCP. *^c^ Missing value: if the data not available, lost to follow-up, or methotrexate was stopped due to inefficacy or adverse effects. ^d^ % of patients of the total available number (excluding the missing value), DAS = Disease activity score, eGFR = Estimated glomerular filtration rate, ESR = Erythrocyte sedimentation rate, MCV = Mean corpuscular volume of erythrocyte.* | | | |

**Supplementary Figure 2: Area Under the Receiver Operating Characteristics (AUROC) curves of MCV change measured at monthly intervals to predict clinical response at six months.**

Bar plots represent AUROC of mean corpuscular volume (MCV) of erythrocyte change of first six months with DAS28-ESR change at three months shown as a comparator. The vertical dashed line represents AUROC >0.70.

**Supplementary Figure 3: Univariate logistic regression.** Odds-ratio (OR) of each of the variables to predict clinical response at six months with 95% CI (95% confidence interval). N = Number of total patients and No/Yes represent non-responders and responders.

*DAS = Disease activity score, eGFR = Estimated glomerular filtration rate, ESR = Erythrocyte sedimentation rate, MCV = Mean corpuscular volume of erythrocyte.*

**Supplementary Table 2: Comparison of multiple logistic regression models to predict clinical response at six months with or without DAS28-ESR or MCV change at 3 months.**

| Variables | N | Multiple logistic regression model | |
| --- | --- | --- | --- |
|  |  | **OR^a^ (95% CI^b^)** | **p-value** |
| Final model without DAS28-ESR change at 3 months (imputed model) ^  *Accuracy ^c^ = 80%, McFadden's R squared = 0.36, AUROC ^d^ = 0.75* | | | |
| MCV change (per 1 fL increase) | 655 | 1.54 (1.39 – 1·70) | <0.001 |
| Concomitant hydroxychloroquine, | Yes = 313, No = 342 | 1·44 (1·02 – 2·04) | 0·037 |
| Antibody status (Seropositive) | Yes = 570, No = 85 | 1·82 (1·08 – 3.07) | 0·023 |
| Age (per 10 years increase) | 655 | 0.92(0·81 – 1·05) | 0·199 |
| Final model without MCV change at 3 months (imputed model) ^e^  *Accuracy ^c^ = 70%, McFadden's R squared = 0.39, AUROC ^d^ = 0.78* | | | |
| DAS28-ESR change (per 1 unit decrease) | 655 | 1.25 (1.19 – 1·30) | <0.001 |
| With hydroxychloroquine, | Yes = 313, No = 342 | 2·73 (1·88 – 3·95) | <0.001 |
| Antibody status (Seropositive) | Yes = 570, No = 85 | 1·73 (1·00 – 2.97) | 0·047 |
| Age (per 10 years increase) | 655 | 0.87(0·76 – 0.99) | 0·041 |
| Final model (unimputed model):  *Accuracy ^c^ = 77%, McFadden's R squared = 0.47, AUROC ^d^ = 0.81* | | | |
| MCV change (per 1 fL increase) | 596 | 1.55 (1.38 – 1·75) | <0.001 |
| DAS28 ESR change (per 1-unit decrease) | 596 | 1.25 (1.19 – 1·31) | <0.001 |
| With hydroxychloroquine, | Yes = 288, No = 308 | 2·18 (1·45 – 3·29) | <0.001 |
| Antibody status (Seropositive) | Yes = 527, No = 69 | 2.25 (1·21 – 4.19) | 0.010 |
| Age (per 10 years increase) | 596 | 0.84(0·75 – 0.97) | 0.019 |
| *^a^ OR = Odds Ratio, ^b^ CI = Confidence Interval.*  *^c^ Accuracy= model accuracy of Cohort 1 (discovery cohort) to predict outcome in Cohort 2 (validation cohort) using the final model.*  *^d^ AUROC= Area Under the Receiver Operating Characteristics curve.*  *^e^ For LASSO missing values were imputed using multivariate imputation by chained equations of the Markov chain Monte Carlo method under the ‘missing at random’ assumption.*  *DAS = Disease activity score, ESR = Erythrocyte sedimentation rate, MCV = Mean corpuscular volume of erythrocyte.* | | | |

**Supplementary Figure 4 (A-B): Performance of various machine learning approaches.** Comparison of (A) Area Under the Receiver Operating Characteristics (AUROC) (B) accuracy of different machine learning approaches using Cohort 1 (Discovery cohort) to predict clinical response at six months in Cohort 2 (Validation cohort).

**Supplementary Figure 5 (A-B): Comparison of the variable importance score of the predictors to predict methotrexate response at six months by (A) support vector machine (SVM) and (B) logistic regression.** Points on the right side indicate favourable response to methotrexate and points on the left indicate negative predictors.

*The SVM model was constructed with Cohort 1 using e1071(3) R package to predict methotrexate response in the validation cohort. Radial basis function kernel-based SVM was used(4), and after 10-fold cross-validation of data, cost = 0.1 was selected as it resulted in the lowest cross-validation error rate in order to obtain the best-tuned model. The importance score of the best-tuned model is shown in (A). For the logistic regression model, vip(5) R package was used to obtain the importance score, shown in (B).*

**Supplementary Figure 6 (A-D): Receiver operating characteristic (ROC) curve to identify the optimal mean corpuscular volume (MCV) threshold at month three to predict clinical response at six months.** (A) ROC of MCV change at three months to predict methotrexate (MTX) response for all patients from Cohort 1 with the distribution of MCV change (B) along with optimal cut-point (indicated by black dot in A and black line in B). (C) ROC curve grouped by MTX monotherapy versus MTX combined with hydroxychloroquine (HCQ) and (D) distribution of MCV change with optimal cut-point stratified by treatment exposure are shown.

# *To select the optimal cut-point of MCV, we used cutpointr (6) R package. After bootstrapping 1000 times, maximize boot metric method was used to identify optimal cut-point of MCV to predict the methotrexate response.*

**
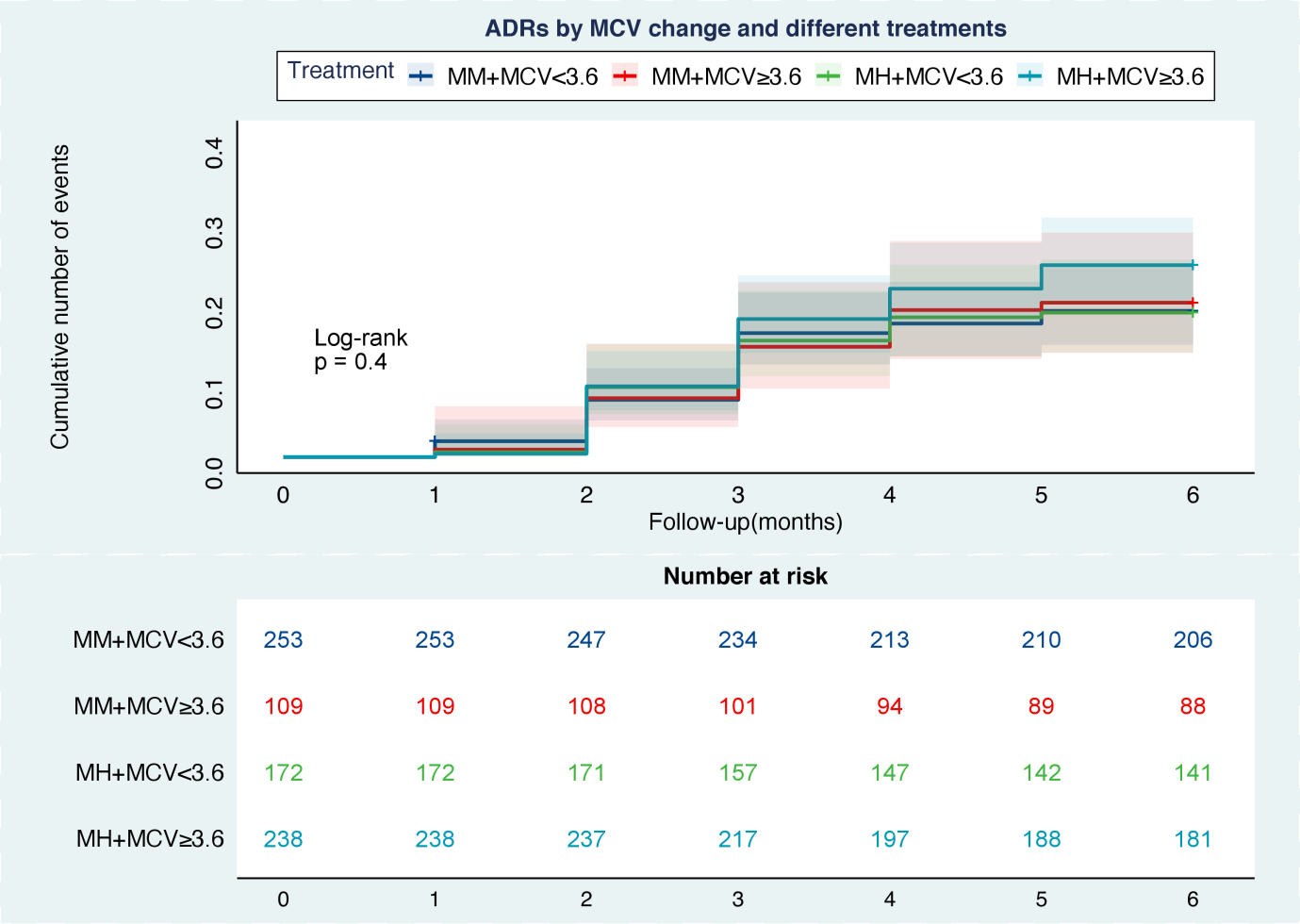
**

**Supplementary Figure 7: Kaplan Meier (K-M) curve of cumulative events of adverse drug reactions (ADRs) in the first six months (both cohorts combined) after initiation of methotrexate stratified by change in mean corpuscular volume (MCV) (<3·6 or ≥3·6 fL) at three months from baseline and concomitant treatment – Methotrexate monotherapy (MM) versus methotrexate with hydroxychloroquine (MH).**

**Supplementary Figure 8: Types of adverse drug reactions (ADRs).** Number within the box at the top of the bars indicates percentage of the patients (both cohorts combined). No significant difference was observed by pairwise non-parametric testing.

**Supplementary Figure 9 (A-B): Changes in mean corpuscular volume after initiation of methotrexate with or without hydroxychloroquine stratified by responder versus non-responder status in Cohort 2.** Linear mixed model was used to estimate mean changes (A) of MCV (mean corpuscular volume of erythrocyte) at each time point from baseline. Mean changes and number of available patients at each time points are shown at the bottom of the plot. (B) Heatmap demonstrating p-values of pairwise comparisons of each of these treatment groups and response status.

*HCQ = hydroxychloroquine, MCV = mean corpuscular volume of erythrocytes, MTX = methotrexate, NR = non-responders, R = responders.*

**

**Supplementary Figure 10: The number of responders and non-responder within the latent classes of cohort 1.**

^$^ Fisher’s exact test.


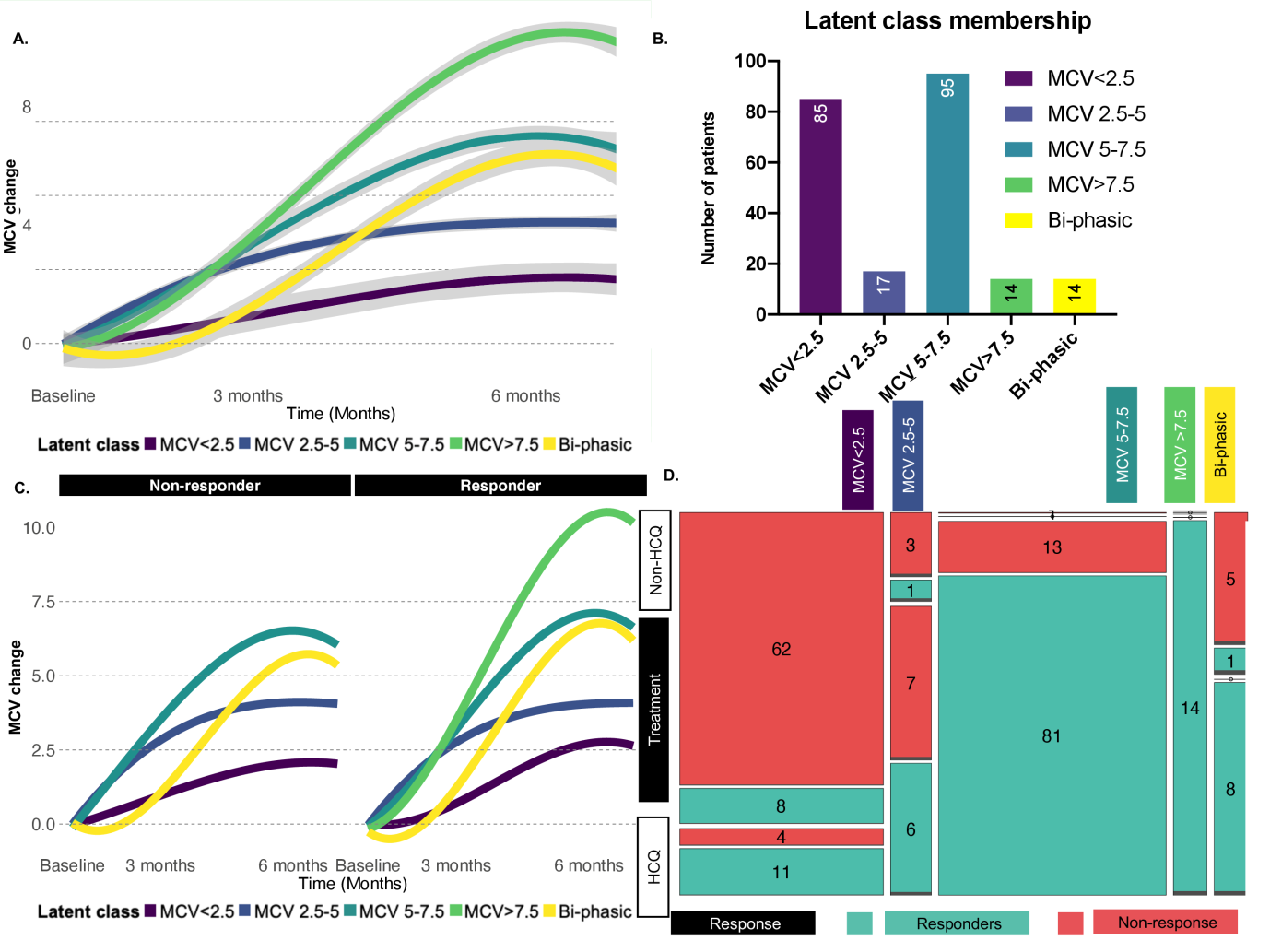


**Supplementary Figure 11 (A-D): Results demonstrating the five-latent class model of erythrocyte mean corpuscular volume (MCV) change over the first six months after initiation of methotrexate generated from Cohort 1 applied to Cohort 2 (Overall model entropy 0.914).** The classes are *1.* ***MCV <2.5:***  *MCV increase less than 2.5 fL from baseline, 2.* ***MCV 2.5-5****: MCV increase between 2.5-5 fL from baseline, 3.* ***MCV 5-7.5****: MCV increase between 5-7.5 fL from baseline, 4.* ***MCV>7.5:*** *MCV increase more than 7.5 fL from baseline, 5.* ***Bi-phasic****: early decrease followed by increase in MCV.* Smoothed curves of the latent class trajectories (A) and the number of patients in each class (B) are shown. The five-class model trajectories stratified by methotrexate responder status are shown in (C). The mosaic plot (D) demonstrating cross-sectional distribution of patients from each latent class stratified by treatment response (responder versus non-responder) and treatment type (concomitant HCQ = hydroxychloroquine versus non-HCQ = no hydroxychloroquine). The widths of the boxes are proportional to the percentage of the patients of each latent class, whereas the height indicates the proportion of patients in each treatment group and responder status. The numbers inside the boxes represent the number of patients in each group.

*A same five-class model derived from Cohort 1 was applied and adjusted for age at baseline, disease duration, gender, ethnicity, methotrexate dose at three months, prednisolone dose at three months, baseline hemoglobin, baseline estimated glomerular filtration rate and concomitant disease modifying agent (methotrexate, hydroxychloroquine or sulfasalazine). Model accuracy was assessed by the average of the maximum posterior probability of assignments (APPA) – 1. MCV<2.5: 0.976, 2.MCV<5 fL: 0.862, 3. MCV<7.5: 0.951, 4. Class >7.5fL: 0.908 and 5. Biphasic: 0.882.*

**References**

1. Cecile Proust-Lima VP, Benoit Liquet . Estimation of Extended Mixed Models Using Latent Classes and Latent Processes: The R Package lcmm. Journal of Statistical Software,. 2017;78(2):1-56.

2. Stef van Buuren KG-O. Mice: Multivariate Imputation by Chained Equations in R. Journal of Statistical Software. 2011;45(3):1-67.

3. Leisch DMaEDaKHaAWaF. e1071: Misc Functions of the Department of Statistics, Probability

Theory Group (Formerly: E1071), TU Wien. R package version 17-4. 2020(<https://CRAN.R-project.org/package=e1071>).

4. Huang M-W, Chen C-W, Lin W-C, Ke S-W, Tsai C-F. SVM and SVM Ensembles in Breast Cancer Prediction. PLoS One. 2017;12(1):e0161501-e.

5. Brandon Greenwell BBaBG. vip: Variable Importance Plots. R package version. 2020; 0.2.2.

6. Thiele C. cutpointr: Determine and Evaluate Optimal Cutpoints in Binary Classification Tasks. R package version 1032. 2020.
